# Femoral Skeletal Growth in Early Infancy Assessed by Radiofrequency Echographic Multi‐Spectrometry

**DOI:** 10.1002/jcu.70261

**Published:** 2026-04-27

**Authors:** Serafina Perrone, Virginia Beretta, Laura Cannavò, Domenico Corica, Tommaso Aversa, Giorgia Pepe, Letteria Morabito, Maria Elisabeth Street, Malgorzata Wasniewska, Tullio Ghi, Andrea Dall'Asta

**Affiliations:** ^1^ Department of Medicine and Surgery, Neonatology Unit, Pietro Barilla Children's Hospital University of Parma Parma Italy; ^2^ Pediatric Intensive Care Unit University Hospital of Verona Verona Italy; ^3^ Department of Human Pathology of Adulthood and Childhood, Unit of Pediatrics University of Messina Messina Italy; ^4^ Department of Medicine and Surgery, Paediatric Clinic, Pietro Barilla Children's Hospital University of Parma Parma Italy; ^5^ Department of Medicine and Surgery, Obstetrics and Gynecology Unit University of Parma Parma Italy; ^6^ Department of Mother and Child, Obstetrics and Gynecology Unit University Hospital of Parma Parma Italy

**Keywords:** bone mineral density (BMD), neonatal bone assessment, osteoporosis prevention, partial least squares (PLS), radiofrequency echographic multi‐spectrometry (REMS), skeletal growth

## Abstract

**Background:**

Early skeletal growth represents a critical window for lifelong bone health and is influenced by genetic, nutritional, and environmental factors. However, the assessment of bone status during infancy remains challenging due to the limited feasibility of reference densitometric techniques for longitudinal use. Radiofrequency echographic multi‐spectrometry (REMS) is an ultrasound‐based technology validated for bone mineral density (BMD) assessment in adults, but its applicability to early infancy has not yet been established. This study aimed to explore a REMS‐based approach for investigating femoral skeletal growth during early life.

**Methods:**

Eighty‐two healthy term infants underwent femoral REMS examinations at 3 and 6 months of age. Unfiltered radiofrequency ultrasound signals were analyzed using a multivariate framework combining fast Fourier transform (FFT) spectral analysis with partial least squares (PLS) regression. Body weight was used as an indirect surrogate marker of skeletal growth, with spectral features serving as predictors.

**Results:**

Average power spectra stratified according to PLS‐inferred body weight demonstrated distinct spectral patterns between lower‐ and higher‐weight infants. The PLS‐based spectral model showed longitudinal consistency, preserving relative skeletal status across time points. These findings indicate that REMS‐derived spectral features vary systematically with growth‐related physiological changes during infancy.

**Conclusions:**

REMS‐derived spectral characteristics appear to be sensitive to early skeletal growth processes in infancy. Although further studies incorporating direct bone outcomes are required to disentangle the relative contributions of body size and mineralization, this work provides foundational evidence supporting the feasibility of extending REMS technology to early life. REMS may represent a promising non‐invasive and radiation‐free approach for longitudinal investigation of skeletal development in pediatric populations.

## Graphical abstract

Radiofrequency echographic multi‐spectrometry (REMS) acquisition of raw ultrasound radiofrequency signals from neonatal bone. Signals undergo preprocessing and frequency‐domain analysis to extract spectral features reflecting bone properties. A software‐guided procedure then identifies and selects standardized regions of interest (ROIs). The resulting spectral profiles are compared with reference models to enable quantitative, reproducible, and non‐invasive assessment of neonatal bone quality.

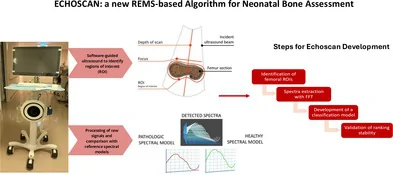

## 1 Introduction

Skeletal status is determined by the amount of bone acquired during skeletal development, which begins in the womb and continues after birth (Perrone et al. 2023). The mechanisms occurring in the intrauterine and postnatal period remain unclear, but skeletal metabolism is thought to be influenced by oxidative stress and exposure to endocrine disruptors (Perrone et al. 2016; Dirkes et al. 2021).

Assessing skeletal status in infants is crucial for early detection of conditions associated with deficient bone mass or mineralization and for monitoring bone development (Cooper et al. 1997; Kim et al. 2017; Boskey and Coleman 2010). Currently, dual x‐ray absorptiometry (DXA) is the most widely employed method for assessing bone mineralization in early life. However, this technique has notable limitations in infants, such as radiation exposure and motion artifacts, which limit its application, particularly in longitudinal studies (Shepherd 2017; Crabtree and Kent 2016).

Bone ultrasonography was historically explored as a non‐invasive, radiation‐free method to assess bone status in neonates and infants. Quantitative ultrasound (QUS) measurements, typically performed at peripheral sites such as the tibia or radius, aimed to provide surrogate markers of bone quality, including parameters like speed of sound (SOS) and broadband ultrasound attenuation (BUA), which may positively correlate with birth weight, length, and gestational age (Ritschl et al. 2005; Gonnelli et al. 2004).

However, despite its theoretical advantages, conventional peripheral QUS has shown limited standardization, reproducibility, and clinical applicability for neonatal and infant bone assessment (Ritschl et al. 2005; Gonnelli et al. 2004; Fewtrell 2003; Szabo et al. 2011). These limitations are related in part to the specific acoustic and structural features of infant bone, including small bone size, high water content, rapid growth and remodeling, and the difficulty of achieving standardized acquisitions in early life. Such constraints have motivated the search for more robust nonionizing approaches for early bone health monitoring.

In this context, the radiofrequency multichannel ultrasound spectrometry (REMS) technique appears to offer both the advantages of the ultrasound technique and good precision and diagnostic accuracy comparable to DXA (Amorim et al. 2021, 2025; Giovanni et al. 2024; Ishizu et al. 2023). To date, the REMS technique is widely used for measuring bone mineral density (BMD) in adults, but no studies have explored its applications in newborns (Diez‐Perez et al. 2019; Di Paola et al. 2019; Cortet et al. 2021; Messina et al. 2023; Ramirez Zegarra et al. 2024; Fuggle et al. 2024).

In this study, we investigate the relationship between REMS‐based ultrasound spectral features and body growth in early infancy, with a focus on longitudinal femoral growth assessment, while explicitly treating the present work as a preliminary exploratory feasibility study rather than a direct validation of infant BMD measurement.

## 2 Methods

This multicenter, longitudinal, prospective study is part of the research project titled “Radiofrequency Echographic Multi‐Spectrometry for Early Bone Health: The REMS‐Bone Study Protocol” (Trial acronym: REMS‐Bone) (Perrone, Wasniewska, et al. 2025). The study was approved by the Institutional Review Board of Parma University Hospital (Ethics Committee of the Emilia Nord Wide Area for protocol ID SIRER 5484 REMS‐BONE. PNRR‐MAD‐2022‐12 376 819). Written informed consent was signed by both parents and all the data were anonymized before being used for the statistical analysis.

### 2.1 Population

From June 2024 to August 2024, 82 healthy term newborns (mean gestational age: 39.5 ± 1.3 weeks, birth weight: 3500 ± 600 g) were consecutively enrolled and followed until 6 months of age. Inclusion criteria were: full‐term newborns from low‐risk pregnancies, absence of current or previous maternal conditions that could potentially interfere with bone metabolism, absence of maternal motor disabilities, no maternal history of recent bone fractures, absence of a diagnosis of osteopenia or osteoporosis according to the criteria of the Italian Society for Osteoporosis, Mineral Metabolism, and Skeletal Diseases (SIOMMMS). Newborns hospitalized from birth for specific conditions, newborns with metabolic disorders, newborns with genetic syndromes were excluded.

### 2.2 Data Acquisition Procedure

**FIGURE 1:**
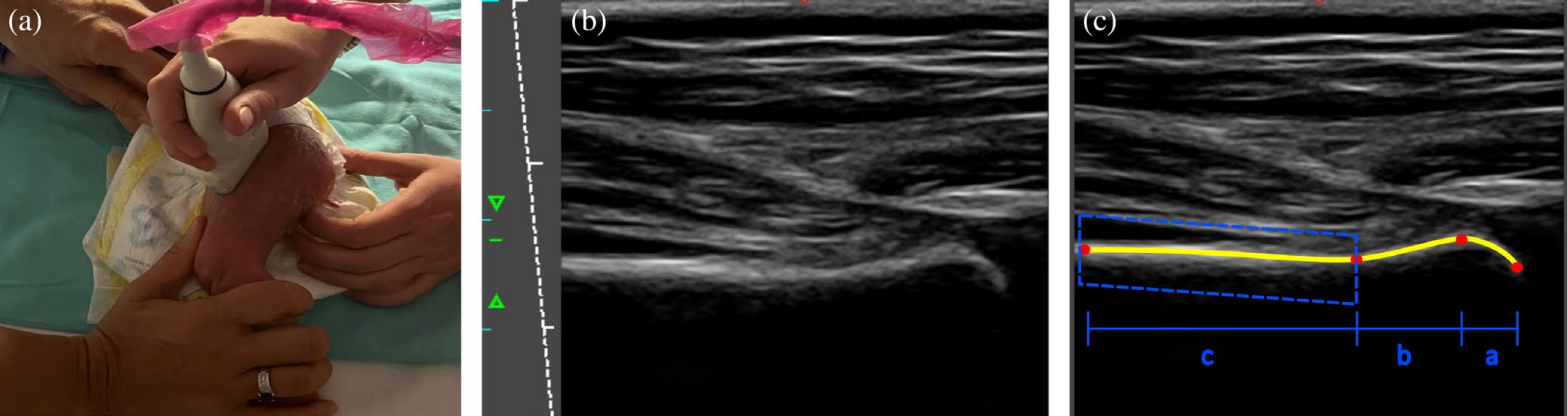
ROI extraction procedure from a B‐mode ultrasound image of the infant's femur. Left: Probe positioning. Center: B‐mode acquisition. Right: Four manually placed red dots reference points, encompassing (a) proximal epiphysis, (b) metaphysis, (c) diaphysis, are used to interpolate the femoral profile (solid yellow line), around which we extract the ROI matrix (dotted blue box) containing radio frequency ultrasound signals from the bone.

REMS scans were performed at femoral sites by employing a dedicated echographic device (EchoStation, Echolight Spa, Lecce, Italy), equipped with a 40 mm linear transducer operating at the nominal frequency of 9 MHz and used as recommended by the manufacturer. The manufacturer provided the device configured for research purposes, which allowed access to the raw data acquired through the scans. During REMS investigations, the linear probe was positioned longitudinally over the infant's femur in order to visualize the target bone interface and the main femoral landmarks. Scan depth and transducer focus were adjusted to visualize the same femoral segment consistently across acquisitions, and five B‐mode frames were acquired for each infant. Radiofrequency (RF) raw signals were simultaneously acquired together with the B‐mode frames (Figure 1).

Regions of interest (ROIs) encompassing femoral diaphysis were manually delineated on the B‐mode images according to a standardized workflow. The selected ROIs were then mapped to the corresponding locations in the raw RF data space, enabling precise extraction of the relevant RF signals. This workflow was designed to maximize consistency of anatomical targeting across examinations, while acknowledging that a dedicated reproducibility analysis was beyond the scope of the present preliminary study.

Since no existing algorithm applies REMS technology to neonatal bone assessment, we developed a novel approach tailored to early infancy, including the following key steps:

1. Identification of the femoral ROI: Each femur was manually delineated to ensure precise localization of the femoral diaphysis of infants at 3 and 6 months. A standardized diaphyseal ROI was defined to optimize bone signal extraction and maintain measurement consistency across subjects.
2. FFT analysis (spectral averaging): Frequency‐based analysis using the Fast Fourier Transform (FFT) was applied to obtain averaged spectral signatures, facilitating the extraction of key bone characteristics.
3. Explanatory model fit: Partial least squares (PLS) regression was fit cross‐sectionally on the entire dataset having spectral features as explanatory variables and infant weights as dependent variables.
4. Model consistency check: PLS expected body weights at 3 and 6 months were used to rank infants at each time‐point, and the consistency of the ranking between time‐points was used to check the stability of the modeling over time.

#### 2.2.1 ROI Identification

Femoral ROIs were identified through a standardized procedure to ensure accurate and reproducible ROI extraction. The process (Figure 1) involved the following steps:

– Linear probe placement: The probe was positioned longitudinally on the femur to visualize the proximal epiphysis, metaphysis, and diaphysis in the same acquisition plane.
– Manual annotation of anatomical landmarks: Four reference points were manually marked on the B‐mode image to identify the proximal epiphysis, metaphysis, and diaphyseal profile and to guide subsequent interpolation.
– Femoral profile interpolation: A smooth representation of the femoral contour was obtained through interpolation of the annotated landmarks.
– ROI matrix creation: A box centered on the diaphysis was selected to contain the RF ultrasound signals used for spectral analysis. The collection of one‐dimensional signals within the ROI was grouped into a matrix for further spectral processing, while maintaining a consistent anatomical target and ROI logic across examinations.

#### 2.2.2 Feature Extraction From ROI

**FIGURE 2:**
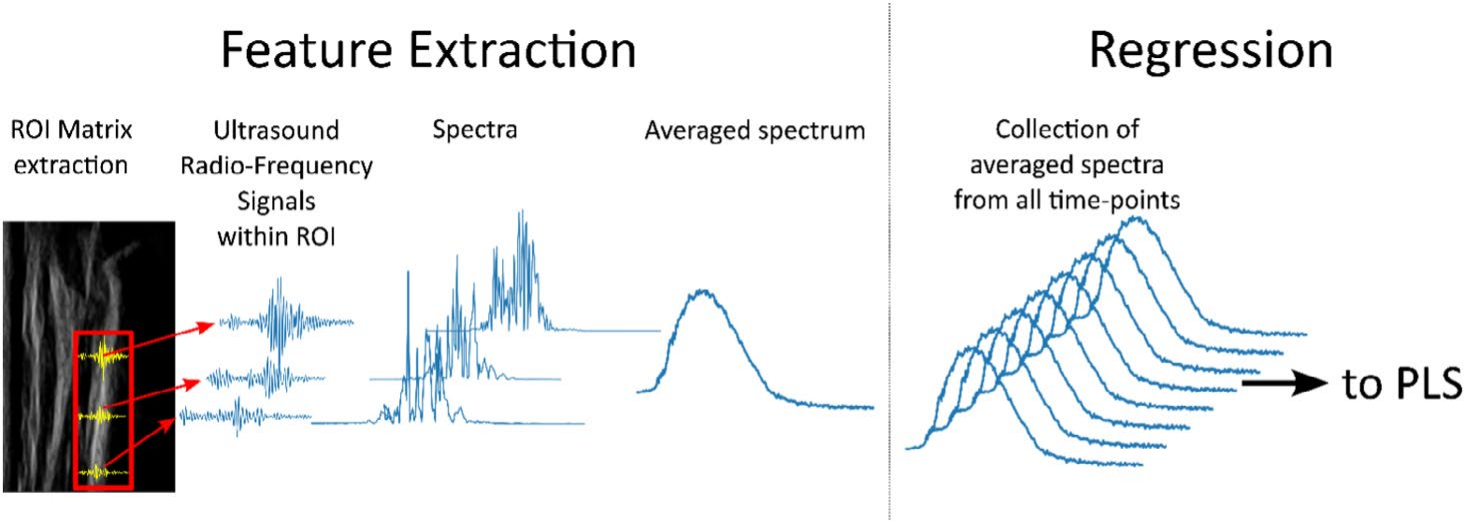
Feature extraction and PLS regression from left to right: (1) ROI matrix identification (cf. Figure 1) in B‐mode images—images are shown rotated by 90° with respect to images in Figure 1; (2) ultrasound radio‐frequency signals extracted from the ROI (only three shown for graphical purposes); (3) spectra computation (no. of bands = 256); (4) spectra average; and (5) PLS regression having as explanatory variable the collection of averaged spectra from all infants at all time‐points.

The selected ROIs were mapped to the corresponding locations in the raw RF data to extract the underlying RF signals (Figure 1). Then, the extracted signals underwent a series of pre‐processing steps to extract spectral features (Figure 2):

- DC component removal: The continuous component of the signal was eliminated to enhance spectral analysis. Additional preprocessing steps included implicit normalization and averaging across multiple RF lines to reduce local variability and random interference effects.
- Power spectrum computation: Each trace underwent spectral FFT decomposition to extract the power distribution across frequencies, resulting in 256 bands spanning from 0 to 20 MHz. The spectra were then averaged over each ROI, resulting in a single spectrum for each infant and each time point. Spectra were lastly normalized in decibels relative to the carrier (dBc) to ensure consistency and comparability across measurements.
- PLS regression: The collection of averaged spectra from all infants at all time points was fed to the PLS modeling routine, described in the next paragraph.

#### 2.2.3 PLS Regression Model

When modeling the relationship between infant body weight and many correlated bone spectral features (256 frequency bands) extracted from the femur diaphysis ROIs, the classic ordinary least squares (OLS) regression model struggles to perform well. This issue is known as collinearity: when predictors are highly correlated it becomes difficult for the model to isolate the individual effect of each variable on the response. Collinearity undermines the stability and interpretability of OLS estimates. PLS was historically developed to overcome this limitation (Wold et al. 1984).

PLS regression is an advanced multivariate linear model designed to efficiently capture relationships between input features and the target variable, without the pitfalls of OLS (Höskuldsson 1988; Wold et al. 2001).

It is based on the geometric projection of the original high‐dimensional feature space (256 frequency bands in our case) into a lower dimensional latent space. We fit the PLS model through the open‐source fitting routine “PLS Regression” in the Sklearn python library (Pedregosa et al. 2011). To avoid overfitting, the dimensionality of the lower dimensional space (“n_components” in the sklearn library) was treated as a hyperparameter to be tuned and optimized through *k*‐fold cross‐validation (Montesinos López et al. 2022; Arlot and Celisse 2010), with *k* = 5. Specifically, the dataset was split into five parts, or folds; for each possible number of latent dimensions (search space from 1 to 10 dims), the PLS model is trained on fourfolds and validated on the fifth. This is repeated across all folds. The number of latent dimensions with the best average validation mean‐squared‐error is chosen as optimal. The resulting optimal latent space was five‐dimensional.

Finally, we fit the PLS regression model by setting a five‐dimensional latent space, using 256 bone spectral features as explanatory variables and body weight as the outcome variable. The model was fit cross‐sectionally on all available infants' data at all time‐points.

Body weight was adopted only as an indirect growth‐related surrogate and not as a direct measure of BMD or mineralization.

To further strengthen the robustness of the analysis, we applied an additional round of fivefold cross‐validation. This allowed us to generate predictions on data not seen during model fitting, thereby reducing overfitting and providing a more reliable estimate of the model's generalization performance.

Because the present study was exploratory and not designed as a predictive clinical tool, an independent external validation set was not included at this stage.

Once fitted, we qualitatively checked the relationship between spectral features and expected body weight by stratifying the data in equally numerous body weight tertiles, and averaging the spectral features over the first tertile (infants with expected low‐weight) and over the third tertile (infants with expected high‐weight).

This whole‐spectrum comparison was intended to explore global spectral differences rather than to derive a single quantitative spectral biomarker.

Moreover, given the expected and observed monotonic relationship between infants' body weights at 3 versus weight at 6 months, we evaluated the longitudinal consistency of the PLS model by examining whether a similar relationship was preserved in the PLS‐inferred weights derived from spectral features as explanatory variables. The strength of the relationship was assessed through Spearman and Kendall rank correlation coefficients (Spearman 1961; Forthofer and Lehnen 1981).

## 3 Results

The study included 82 full‐term newborns. Clinical characteristics of the study population are reported in Table 1.

**TABLE 1:** Auxological characteristics of the study population.

	Mean ± SD	Minimum	Maximum
Gestational age at birth (weeks)	39.5 ± 1.3	37	41
Length at birth (cm)	49.9 ± 1.6	48	54
Length at 3 months of age	62.1 + 2.6	56	69
Length at 6 months of age	68.8 ± 2.8	52	73
Weight at birth (kg)	3.5 ± 0.6	2.7	4.4
Weight at 3 months of age	5.9 ± 0.8	4.6	7.1
Weight at 6 months of age	7.9 ± 1.1	6.5	9.5
Head circumference at birth (cm)	34.6 ± 0.9	33	36
Head circumference at 3 months of age	41.3 ± 1.3	37	45
Head circumference at 6 months of age	44.4 ± 1.6	40	49

**FIGURE 3:**
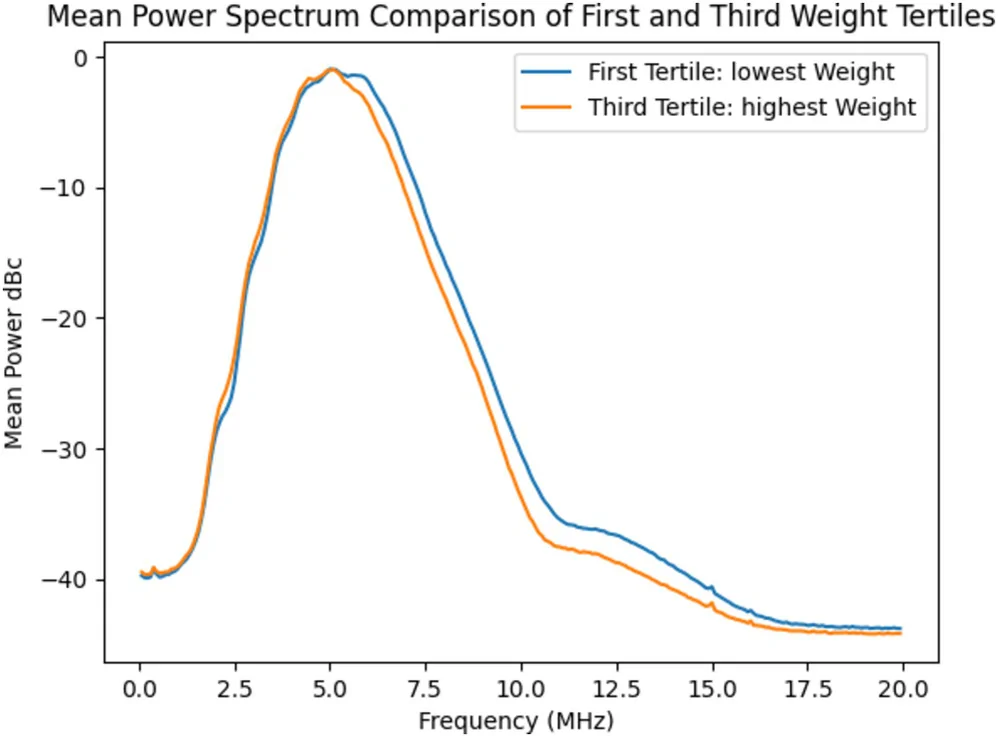
Mean power spectrum comparison of first and third weight tertiles. If we assume weight as a surrogate proxy for bone mineral density, observed spectral separation is consistent with previously reported findings on adults (Conversano et al. 2015).

Spectral measurements were associated with body weight growth of infants (Figure 3). Specifically, when the spectral data is stratified into tertiles based on infant body weight, we observe a clear pattern: the average spectrum corresponding to the lowest weight tertile consistently lies above the average spectrum of the highest weight tertile.

In line with the exploratory design of the study, this result should be interpreted as evidence of an association between spectral shape and growth‐related variation rather than as direct evidence of bone mineralization measurement.

To evaluate the longitudinal consistency of the PLS model, which we recall was fitted cross‐sectionally, we compared infant body weights at 3 and 6 months using both actual measurements and values inferred by the model from spectral features.

**FIGURE 4:**
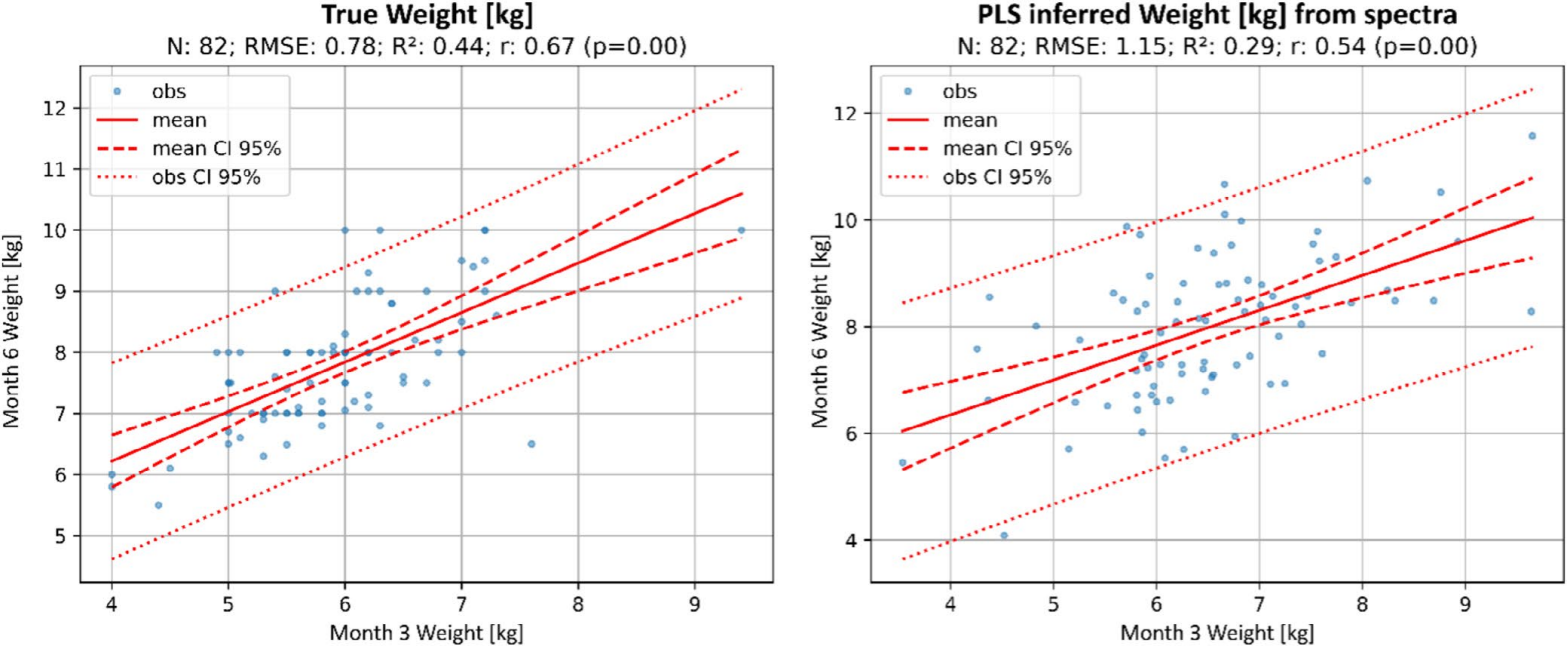
PLS longitudinal model consistency. The left panel shows the relationship between true weights at the two time points, while the right panel shows the same relationship based on PLS‐inferred weights. Left: Monotonic relationship between infants' body weights at 3 and 6 months (Spearman rank correlation = 0.64 *p* < 0.001; Kendall rank correlation = 0.5, *p* < 0.001). Right: Same relationship with PLS inferred body weights from spectra (Spearman rank correlation = 0.49 *p* < 0.001; Kendall rank correlation = 0.34, *p* < 0.001).

A strong monotonic relationship was observed for the true weights (Spearman rank correlation = 0.64, *p* < 0.001; Kendall rank correlation = 0.5, *p* < 0.001) (Figure 4). The presence of a similar trend in the inferred weights supports the internal longitudinal consistency of the model over time. This was statistically significant (Spearman rank correlation = 0.49, *p* < 0.001; Kendall rank correlation = 0.34, *p* < 0.001), although these findings should be interpreted within the exploratory and non‐predictive framework of the present study.

## 4 Discussion

Bone health is of primary importance from birth, as it influences overall growth, development, and long‐term quality of life. Although the mechanisms regulating skeletal development during the intrauterine and early postnatal periods are not yet fully understood, bone metabolism is known to be affected by factors such as oxidative stress and exposure to endocrine disruptors (Perrone et al. 2016; Dirkes et al. 2021). During pregnancy, the fetus acquires most of its calcium through maternal bone resorption, making early skeletal development particularly vulnerable to alterations in mineral homeostasis. Evidence from the literature indicates that osteopenia is increasingly recognized in infants with low birth weight, who are at higher risk of impaired skeletal growth and reduced bone mineralization compared to infants born appropriate for gestational age (Godfrey et al. 2001; Weiler et al. 2002; Lee et al. 2012). During the first months of life, skeletal growth represents a highly dynamic process, modulated by genetic, nutritional, and environmental determinants (Perrone et al. 2023; Fecher‐Trost et al. 2019).

In support of this biological framework, we have previously investigated the role of perinatal oxidative stress in early skeletal development in a longitudinal cohort of healthy term infants using REMS technology. In that preliminary study, markers of oxidative balance measured in cord blood were significantly associated with neonatal anthropometric parameters and with REMS‐derived bone quality indices during the first year of life. Specifically, increased markers of oxidative damage were inversely associated with REMS Z‐scores at later time points. These findings suggest that prenatal redox status plays a relevant role in early skeletal development and, importantly, that REMS‐derived parameters are sensitive to biologically meaningful determinants of bone quality beyond simple size‐related measures (Perrone, Carloni, et al. 2025).

The concept of “bone health” refers to the structural and mechanical properties of bone tissue, which are commonly assessed using imaging techniques aimed at quantifying BMD (Cannalire et al. 2024). However, the application of standard densitometric techniques in early infancy is limited by practical and ethical constraints, particularly due to the use of ionizing radiation. For this reason, alternative, non‐invasive approaches capable of capturing growth‐related skeletal changes are of particular clinical interest in pediatric populations.

In this study, we applied a novel REMS‐based approach using unfiltered native ultrasound signals—namely RF ultrasound signals—acquired during femoral ultrasound examinations and analyzed through advanced multivariate modeling. Given the well‐documented association between body weight and bone mineralization in early life (Godfrey et al. 2001; Weiler et al. 2002; Lee et al. 2012), body weight was used in this study as a surrogate marker, rather than a direct measure, of skeletal mineral status. It is important to emphasize that body weight does not represent BMD; rather, it was employed as an indirect proxy to explore whether ultrasound spectral features are sensitive to physiological changes associated with skeletal growth.

Our primary aim was to investigate the relationship between ultrasound spectral characteristics and early growth patterns, with particular attention to femoral development. The observed relationship between spectral features and body weight manifests as a clear stratification of spectra (Figure 3), whereby spectra corresponding to lower‐weight infants tend to lie above those associated with higher‐weight infants. This systematic separation suggests that ultrasound spectral profiles vary consistently with growth‐related physiological changes. Although body weight cannot be equated with BMD, this finding does not discount the hypothesis that spectral features extracted from RF signals may capture.

These findings may be viewed in light of previous REMS studies in adults, in which spectral stratification was observed according to BMD values, with spectra from individuals with lower BMD positioned above those from individuals with higher BMD (Conversano et al. 2015). This analogy does not validate the current pediatric approach against DXA, nor does it demonstrate direct measurement of infant mineralization; rather, it supports the methodological plausibility of extending an RF‐spectrum‐based framework already established in adults to a preliminary pediatric setting.

REMS technology has already demonstrated effectiveness in the assessment of osteoporosis and in predicting fragility fracture risk in adult populations, with diagnostic accuracy comparable to DXA (Amorim et al. 2021, 2025; Giovanni et al. 2024; Ishizu et al. 2023; Ramirez Zegarra et al. 2024; Fuggle et al. 2024). Compared with other densitometric techniques, REMS offers the additional advantage of providing qualitative information related to bone quality, thereby improving the overall characterization of skeletal status (Pepe et al. 2025; Pisani et al. 2023). Its low cost, portability, ease of use, and most importantly, nonionizing nature make REMS particularly suitable for longitudinal investigations and pediatric applications (Al Refaie et al. 2023).

Potential future applications of a dedicated infant REMS framework may include longitudinal follow‐up of populations at risk for impaired skeletal development, such as preterm infants, infants with suspected metabolic bone disease of prematurity, or infants with nutritional conditions affecting bone health.

Despite these advantages, REMS—like other localized imaging techniques—does not allow for whole‐body skeletal assessment. In the present study, the femoral diaphysis was selected as the anatomical site of interest because of its central role in skeletal growth and mechanical loading during early development. Furthermore, previous studies in adults have demonstrated a strong agreement between REMS and DXA measurements at the femoral site (Di Paola et al. 2019; Cortet et al. 2021; Cannalire et al. 2024). Considering the preliminary nature of this research and the potential risks associated with ionizing procedures, direct BMD measurements were not included at this stage. Consequently, the direct relationship between ultrasound spectral features and BMD, as well as the extent to which these features are independent of body weight, remains to be elucidated and will be the focus of future investigations.

The main limitations of this study arise from its exploratory and non‐predictive statistical framework. The analysis is primarily based on qualitative interpretation of experimental data modeled through regression techniques, rather than on outcome‐driven prediction.

In addition, the study does not include direct validation against a recognized densitometric or biochemical gold standard, and a dedicated intra‐ or inter‐operator reproducibility analysis was not performed. We also acknowledge that the intervening soft tissues between probe and bone may influence ultrasound attenuation and scattering and may therefore contribute to the observed RF spectral variability. To address part of this variability, a standardized acquisition and ROI‐selection workflow was adopted, together with spectral averaging, normalization, and cross‐validation‐based modeling; however, these measures cannot demonstrate complete removal of soft‐tissue‐related contributions.

Overall, the present findings support the potential of REMS as a valuable tool for investigating skeletal development during early life. The development of a REMS‐based algorithm specifically tailored to infancy represents a significant first step toward extending this technology to the pediatric population. Future studies incorporating direct BMD measurements, larger longitudinal datasets and dedicated reproducibility analyses will be essential to validate and refine this approach, ultimately enabling a more comprehensive and quantitative assessment of bone health in early childhood.

## Author Contributions

**Serafina Perrone:** conceptualization, funding acquisition, project administration, resources, validation, supervision, visualization, writing – original draft, writing – review and editing. **Malgorzata Wasniewska:** project administration, supervision, methodology, software, writing – review and editing. **Virginia Beretta:** project administration, investigation, methodology, formal analysis, data curation, writing – original draft, writing – review and editing. **Laura Cannavò:** investigation, methodology, formal analysis, data curation, writing – original draft, writing – review and editing. **Domenico Corica:** supervision, writing – review and editing. **Tommaso Aversa:** supervision, writing – review and editing. **Giorgia Pepe:** investigation, data curation, writing – review and editing. **Letteria Morabito:** investigation, data curation, writing – review and editing. **Maria Elisabeth Street:** supervision, visualization, writing – review and editing. **Andrea Dall'Asta:** investigation, supervision, methodology, writing – review and editing. **Tullio Ghi:** conceptualization, validation, supervision.

## Funding

PNNR‐MAD‐2022‐12376819 project funded under the National Recovery and Resilience Plan (NRRP, M6/C2_CALL 2022 Italian Ministry of Health funded by the European Union—NextGenerationEU). The funder had no role in study design, data analysis, data interpretation, manuscript writing, or the decision to submit the manuscript.

## Conflicts of Interest

The authors declare no conflicts of interest.

## Data Availability

The data that support the findings of this study are available from the corresponding author upon reasonable request.
